# Tweeters, Woofers and Horns: The Complex Orchestration of Calcium Currents in T Lymphocytes

**DOI:** 10.3389/fimmu.2015.00234

**Published:** 2015-05-21

**Authors:** Lilian L. Nohara, Shawna R. Stanwood, Kyla D. Omilusik, Wilfred A. Jefferies

**Affiliations:** ^1^Michael Smith Laboratories, University of British Columbia, Vancouver, BC, Canada; ^2^Department of Microbiology and Immunology, University of British Columbia, Vancouver, BC, Canada; ^3^Centre for Blood Research, University of British Columbia, Vancouver, BC, Canada; ^4^The Djavad Mowafaghian Centre for Brain Health, University of British Columbia, Vancouver, BC, Canada; ^5^Department of Medical Genetics, University of British Columbia, Vancouver, BC, Canada; ^6^Department of Zoology, University of British Columbia, Vancouver, BC, Canada

**Keywords:** calcium, T cell, calcium channels, L-type calcium channels, T cell signaling

## Abstract

Elevation of intracellular calcium ion (Ca^2+^) levels is a vital event that regulates T lymphocyte homeostasis, activation, proliferation, differentiation, and apoptosis. The mechanisms that regulate intracellular Ca^2+^ signaling in lymphocytes involve tightly controlled concinnity of multiple ion channels, membrane receptors, and signaling molecules. T cell receptor (TCR) engagement results in depletion of endoplasmic reticulum (ER) Ca^2+^ stores and subsequent sustained influx of extracellular Ca^2+^ through Ca^2+^ release-activated Ca^2+^ (CRAC) channels in the plasma membrane. This process termed store-operated Ca^2+^ entry (SOCE) involves the ER Ca^2+^ sensing molecule, STIM1, and a pore-forming plasma membrane protein, ORAI1. However, several other important Ca^2+^ channels that are instrumental in T cell function also exist. In this review, we discuss the role of additional Ca^2+^ channel families expressed on the plasma membrane of T cells that likely contribute to Ca^2+^ influx following TCR engagement, which include the TRP channels, the NMDA receptors, the P2X receptors, and the IP_3_ receptors, with a focus on the voltage-dependent Ca^2+^ (Ca_V_) channels.

Immune cells, including T lymphocytes that express a diverse T cell receptor (TCR) repertoire, are key mediators of immune responses against pathogens. T cell activation occurs when its TCR recognizes cognate antigen presented on major histocompatibility complex (MHC) molecules by an antigen presenting cell, which in turn triggers a series of signaling events including calcium (Ca^2+^) signaling. In T cells, elevation of intracellular Ca^2+^ levels is a vital event that mediates T cell activation, proliferation, development, differentiation, homeostasis, effector function, and cell death (1, 2). TCR engagement elicits the activation of tyrosine kinases and subsequently of phospholipase Cγ1 (PLCγ1), which cleaves phosphatidylinositol 4,5-bisphosphate (PIP_2_) from plasma membrane phospholipids to generate diacylglycerol (DAG) and inositol 1,4,5-trisphosphate (IP_3_). DAG, along with Ca^2+^, activates protein kinase C (PKC). IP_3_ binds to IP_3_ receptors (IP_3_R) in the endoplasmic reticulum (ER), leading to the release of Ca^2+^ from the ER intracellular stores into the cytoplasm. It is proposed that as the Ca^2+^ concentration in the ER stores decreases, the process termed store-operated Ca^2+^ entry (SOCE) is triggered, which results in a sustained influx of extracellular Ca^2+^ through Ca^2+^ release-activated Ca^2+^ (CRAC) channels in the plasma membrane (3).

In T cells, several signaling molecules can be activated by Ca^2+^, including the serine/threonine phosphatase calcineurin and its transcription factor target nuclear factor of activated T cells (NFAT), Ca^2+^-calmodulin-dependent kinase (CaMK) and its target cyclic AMP-responsive element-binding protein (CREB), and myocyte enhancer factor 2 (MEF2) targeted by both calcineurin and CaMK, NFκB, and Ras/MAPK pathways (4, 5). In the well-studied calcineurin-NFAT pathway, increased Ca^2+^ levels promote the binding of Ca^2+^ to calmodulin, allowing calmodulin to bind to the serine/threonine phosphatase calcineurin. The activated calcineurin dephosphorylates NFAT resulting in the transport of the dephosphorylated NFAT into the nucleus. NFAT then acting in concert with other transcription factors promotes the integration of signaling pathways and induces differential gene expression patterns dependent on the context of the TCR signaling (2, 6, 7).

Several families of channels expressed on the plasma membrane of T cells contribute to Ca^2+^ influx following TCR engagement (8, 9). ORAI1, the pore-forming plasma membrane subunit of the CRAC channel (10–12), and stromal interaction molecule 1 (STIM1), the ER Ca^2+^ sensing molecule (13, 14), have been proposed to be the major players in the SOCE pathway leading to Ca^2+^ influx from the extracellular space. However, despite the well-established roles of ORAI1 and STIM1 in lymphocyte function, several other important Ca^2+^ channels have been shown to be instrumental in T cell biology (9). The entry of Ca^2+^ in activated T cells can be regulated by transient receptor potential (TRP) channels. Ionotropic glutamate receptors, such as the *N*-methyl-d-aspartate (NMDA) receptors, function as Ca^2+^ channels at the plasma membrane of T cells. Ca^2+^ can also enter the T cell through purinergic P2X receptors, which are channels that become activated once they are bound by their ligand adenosine triphosphate (ATP). IP_3_ receptors (IP_3_R) represent another type of Ca^2+^ channel located at the plasma membrane of T cells. Furthermore, increasing evidence demonstrates the requirement for voltage-dependent Ca^2+^ (Ca_V_) channels, the focus of this review, in T cell Ca^2+^ signaling and function (9, 15–17).

## ORAI and STIM

A well-characterized model of the coordinated action between the pore-forming plasma membrane protein ORAI1 and the ER Ca^2+^ sensor STIM1 has been established (3, 18, 19). Depletion of the ER Ca^2+^ stores following TCR engagement results in oligomerization of STIM1 molecules (20, 21). STIM1 oligomers then accumulate in puncta in regions of the ER beneath the plasma membrane (13, 22, 23), where they directly interact with ORAI1 at the plasma membrane resulting in Ca^2+^ influx from the extracellular space (24, 25).

Evaluation of ORAI1 and STIM1 deficiency in human patients and in mouse models confirmed their physiological role in T cell activation. Loss of functional ORAI1 or STIM1 in humans leads to severe combined immunodeficiency (SCID) (10, 26–30). These patients have normal lymphocyte numbers; however, their T cells show impaired proliferation and cytokine production upon activation as a result of defective SOCE. Similarly to the phenotype observed in humans, *ORAI1^-/-^* and *STIM1^-/-^* mice appear to have normal thymic development of conventional TCRαβ^+^ T cells. However, STIM1- and STIM2-deficient mice have hindered selection of agonist-selected T cells (31). Furthermore, STIM1-deficient T cells have impaired CRAC channel function and subsequent NFAT activation, leading to defective cytokine secretion and T cell responses (32, 33). ORAI1 deficiency in T cells also results in partial reduction in SOCE and cytokine secretion (34, 35). Interestingly, in SCID patients, STIM1 deficiency is also associated with lymphoproliferative and autoimmune diseases (30). This autoimmunity is proposed to be a result of the decreased T_reg_ cell numbers (30, 36). Analogous phenotypes are observed in STIM1- and STIM2-deficient mice (32). It has been suggested that reduced Ca^2+^/NFAT-dependent induction of Foxp3 expression leads to the T_reg_ deficiency (32, 37, 38). Taken together, these findings emphasize the relevance of ORAI1 and STIM1 in T cell function.

The ORAI1 homologs, ORAI2 and ORAI3, which differ in their pharmacology, ion selectivity, activation kinetics, and inactivation properties in comparison to ORAI1, have also been shown to be expressed in T cells (39). Interestingly, while naïve T cells show high levels of ORAI2, its expression is downregulated upon activation, suggesting that ORAI2 may be critical for development or peripheral homeostasis (34, 35). ORAI3 has been shown to form pentamers with ORAI1 to make up the arachidonate-regulated Ca^2+^-selective (ARC) channels (40). However, the role of these arachidonic acid-activated channels in T cells is still poorly understood.

Additionally, naïve T cells express low levels of STIM2, which is upregulated upon TCR activation (41, 42). STIM2 was shown to function as an ER Ca^2+^ sensor and mediate SOCE in lymphocytes, similarly to STIM1. However, studies demonstrate that STIM2 remains active at higher intracellular Ca^2+^ levels than STIM1 (5), and its overexpression only partially rescues Ca^2+^ influx deficiency in STIM1^-/-^ T cells, indicating that STIM2 plays a non-redundant role in these cells (32, 43).

Although the CRAC channel has been the subject of many Ca^2+^ studies concerning T cells, this model does not account for the participation of additional plasma membrane Ca^2+^ channels that have been shown to be expressed and function in T cells. It also does not incorporate the notion that different Ca^2+^ channels may be expressed in specific T cell subsets contributing to differential patterns in Ca^2+^ response (44–46), and ultimately to distinct functional outcomes following TCR engagement. Therefore, it is essential to integrate multiple Ca^2+^ channels into a comprehensive model that takes into consideration the tightly controlled orchestration of these Ca^2+^ channels during Ca^2+^ signaling in T cells.

## TRP Channels

Twenty-eight TRP channel proteins have been identified in mammals, and they are classified based on similarities in amino acid sequence: the classical TRPs (TRPCs), the vanilloid receptor TRPs (TRPVs), the melastatin TRPs (TRPMs), the mucolipins (TRPMLs), the polycystins (TRPPs), and the ankyrin transmembrane protein 1 (TRPA1) (47, 48). The TRP channels form pores that allow cations including Ca^2+^ to pass through (49). Several TRP channel family members can be found in T cell lines or primary T cells (5, 50–52).

Interestingly, TRP channels were evaluated as potential candidates for the CRAC channel prior to the discovery of ORAI1 and STIM1. The TRPV6 channel, which is highly permeable to Ca^2+^, was shown to be induced by store depletion (53). When a dominant-negative pore-region mutant of TRPV6 was expressed in Jurkat T cells, it was found that the CRAC current was reduced (53). However, the role of TRPV6 as a CRAC channel could not be established (54, 55), since BTP2, a CRAC channel inhibitor, did not show an effect on the activity of the TRPV6 channel (56–58).

The TRPC3 channels were also another possible candidate for the CRAC channel due to the evidence that Jurkat T cell lines with mutated TRPC3 channels showed decreased Ca^2+^ influx after TCR stimulation. This could be overcome by wild type TRPC3 overexpression (59, 60). In addition, knockdown of TRPC3 expression in human T cells by siRNA led to diminished proliferation after TCR activation (51). It is important to note that while it has been demonstrated that TRPC3 is activated in response to store depletion (61), DAG seems to be the main stimulus gating TRPC3 (62). Interestingly, in a model of inflammation, a recent study demonstrated that TRPC3 and TRPC6 expression is upregulated in T cells from rats with sepsis (63).

The TRPM2 channel in T cells has also been investigated for its contribution in T cell function. TRPM2, a non-selective cation channel, is induced by the intracellular secondary messengers nicotinamide adenine dinucleotide (NAD^+^), hydrogen peroxide (H_2_O_2_), ADP-ribose (ADPR), and cyclic ADPR (64–66). Studies have suggested that T cell activation can upregulate endogenous ADPR levels in T cells, which results in Ca^2+^ entry via TRPM2 and induction of cell death, indicating that TRPM2 is capable of contributing to Ca^2+^ signaling in T cells (67). The TRPM2 channels have also been associated with T cell effector function. CD4^+^ T cells from TRPM2-deficient mice exhibit impaired ability to proliferate and secrete cytokines after TCR activation. TRPM2-deficient mice also show less inflammation and demyelinating spinal cord lesions in an experimental autoimmune encephalomyelitis model (68).

TRPV1 is an ion channel most well-known for its role as a pain receptor in sensory neurons. It is also known as the vanilloid receptor 1 or the capsaicin receptor, capsaicin being the active ingredient in chili peppers. TRPV1 also has a role in the detection and regulation of body temperature. Recently, Bertin et al. have provided clear evidence that TRPV1 is functionally expressed in CD4^+^ T cells. TRPV1, acting as a non-store-operated Ca^2+^ channel in CD4^+^ T cells, was shown to be critical for TCR-induced Ca^2+^ mobilization, downstream TCR signaling, and cytokine production. By using *in vivo* models of inflammatory bowel disease, a cell-intrinsic role of TRPV1 in promoting the activation and inflammatory responses of T cells was demonstrated (52). Furthermore, a recent study suggested a role for TRPV2, a mechanosensitive channel, during T cell Ca^2+^ signaling (69). Although important to T cell function, the specific functions of TRP channels in Ca^2+^ signaling have yet to be fully explored.

## NMDA Receptors

Glutamate receptors are typically categorized as being metabotropic or ionotropic. The latter category includes the AMPA, kainate, and NMDA receptors (70). The NMDA receptors are a class of ligand-gated glutamate ionotropic receptors typically found in the central nervous system that play a crucial role in neuronal function. The subunits of the NMDA receptor are called NR1, NR2, and NR3 (71). The NMDA receptor acts as an ion channel that is highly permeable to K^+^, Na^+^, and Ca^2+^ (72). The NMDA receptor is activated by glycine and glutamate, consequently resulting in Ca^2+^ influx (72). Various studies have confirmed that NMDA receptor subunits are expressed in human, rat, mouse, and rabbit lymphocytes (73–75). NMDA receptors have been shown to contribute to the increase in intracellular Ca^2+^ levels following T cell activation (73, 74, 76–79). In addition, it has been proposed that NMDA receptor-mediated increase in intracellular Ca^2+^ results in activation of Ca^2+^-dependent PKC, increase in reactive oxygen species (ROS) levels, and subsequent induction of either necrotic or apoptotic cell death in lymphocytes (80). Studies suggest that NMDA receptors participate, at least to some degree, in SOCE, as an NMDA receptor antagonist did not affect the thapsigargin-induced Ca^2+^ release from the ER intracellular stores in T cells, but reduced the influx of Ca^2+^ from the extracellular space (81). Interestingly, NMDA receptors were shown to localize to the immunological synapse following TCR engagement in thymocytes (82). In this scenario, NMDA receptors on the T cells are activated by glutamate released by dendritic cells (DCs), triggering a sustained Ca^2+^ response. It is proposed that this pathway may contribute to negative selection in the thymus by inducing apoptosis in thymocytes, while it may influence proliferation in peripheral T cells (82). The NMDA receptor has additionally been linked to T cell cytokine production and T cell proliferation (83). It has been shown that CD4+ T cells treated with anti-CD3 and ifenprodil, an antagonist that targets the NMDA receptor subunit GluN2B (NR2B), exhibit diminished proliferation (83). Additional research will greatly elevate our current knowledge of the role of the NMDA receptors in shaping the Ca^2+^ signal in T cells.

## P2X Receptors

The P2X receptors are ion channels that facilitate the influx of Ca^2+^ and Na^+^ ions and the efflux of K^+^ ions in response to ATP binding (84). Several subunits of the P2X receptor have been shown to be expressed in human T cells, including P2X1, P2X4, P2X5, and P2X7 (85, 86). Upon TCR engagement, ATP is released through Pannexin 1 hemichannels that localize to the immunological synapse. When liberated, ATP acts on the P2X channels to promote Ca^2+^ influx and enhance signaling (85, 87, 88). Woehrle et al. demonstrated that human T cells exhibit impaired Ca^2+^ signaling after anti-CD3 treatment, when P2X1, P2X4, and P2X7 are inhibited (86). In addition, there is evidence indicating that P2X1, P2X4, and P2X7 contribute to the increase in intracellular Ca^2+^, NFAT activation, proliferation, and IL-2 production in murine and human T cells following stimulation (85, 86, 88, 89). A recent study by Abramowski et al. showed that activation of T cells leads to greater expression of P2X5. Furthermore, P2X5 has been tied to T cell cytokine production, particularly IL-10 (90), while the P2X7 receptor is involved in the secretion of IL-1β, IL-10, and IL-18 by immune cells (84). Additionally, in two models of T cell-dependent inflammation, treatment with a P2X receptor antagonist impeded the development of colitogenic T cells in inflammatory bowel disease, and induced unresponsiveness in anti-islet TCR transgenic T cells in diabetes (88). It is also known that C57BL/6 mice carry a P2X7 P451L mutation, whereas this mutation is not found in Balb/c mice (91). Interestingly, levels of IL-2 production by activated Balb/c lymphocytes are higher compared to those of activated C57BL/6 lymphocytes, further delineating a role for P2X receptors in T cell function (85). P2X7 mutations also exist in humans in the form of single nucleotide polymorphisms (SNPs), which have been linked to conditions such as major depressive disorder, bipolar disorder, and chronic lymphocytic leukemia (84). Human T cells have been shown to undergo shedding of CD62L in response to ATP stimulation (92). However, this process is hindered in human T cells that are homozygous for the P2X7 SNP Glu496Ala, indicating an important role for P2X7 in this context (92). Although many studies have demonstrated the importance of the P2X receptors in T cells, more integrative analysis is required in order to fully appreciate their connections with other Ca^2+^ channel families.

## IP_3_ Receptors

Although IP_3_ receptors (IP_3_Rs) have been well-characterized in the ER, some evidence demonstrates that they may also exist at the plasma membrane of T lymphocytes (8, 93). It is thought that Ca^2+^ channels at the cell surface induced by IP_3_ might only play a role in short-term Ca^2+^ signaling, since IP_3_ dissipates swiftly following TCR engagement (8). It was also proposed that ER IP_3_Rs, which bind IP_3_ to release Ca^2+^ from ER stores, undergo a conformational change upon depletion of ER stores, and signal to surface IP_3_Rs to open (94). Cultured T cells have been shown to have IP_3_Rs on their cell surface (93, 95); yet, Ca^2+^ currents across the plasma membrane induced by IP_3_ failed to be observed (96). It has also been suggested that IP_3_Rs function at the plasma membrane as scaffolds, based on the multiple protein binding sites found in the modulatory domain of the channel (97). Additional research is needed in order to shed more light on the role of the plasma membrane IP_3_Rs in T cell Ca^2+^ signaling.

## Ca_V_ Channels

As their name suggests, the voltage-dependent Ca^2+^ channels, or Ca_V_ channels, enable the influx of Ca^2+^ ions following changes in membrane potential, specifically depolarization (98). For this reason, Ca_V_ channels have traditionally been associated with excitable cells (99). For example, in muscle, some Ca_V_ channels are known to play a role in excitation–contraction coupling (98). The Ca_V_ channels can be designated as high voltage-activated (HVA) or low voltage-activated (LVA) (100). They can also be split into the following types: L (long-lasting and large)-type, P/Q (Purkinje)-type, N (neuronal)-type, R (toxin-resistant)-type, and T (transient and tiny)-type (100).

The Ca_V_ channels structurally consist of the α_1_, β, γ, and α_2_δ subunits, the latter being the result of connective disulfide bonds between the α_2_ and δ subunits (99). The α_1_ subunit of the Ca_V_ channel forms the pore and it is responsible for the channel’s unique properties, whereas the other subunits regulate the structure and activity of α_1_ (99). Four homologous repeated motifs (I–IV), each with six transmembrane segments (S1–S6) and a re-entrant pore-forming loop (P-loop) between S5 and S6, make up the α_1_ subunit. The P-loop contains four highly conserved negatively charged amino acids responsible for selecting and conducting Ca^2+^, while the S6 segments form the inner pore (99). The S4 segments are positively charged and constitute the voltage sensor. The opening and closing of the pore occur via voltage-mediated movement of this sensor (100).

There exist ten α_1_ subunits in mammals, and these can be grouped according to similarities in amino acid sequence. The Ca_V_1 family (Ca_V_1.1–Ca_V_1.4) contains L-type channels; the Ca_V_2 family consists of P/Q-type (Ca_V_2.1), N-type (Ca_V_2.2), and R-type (Ca_V_2.3) channels; and the Ca_V_3 family (Ca_V_3.1–Ca_V_3.3) is also referred to as the T-type channels (99). Many pharmacological and genetic studies have provided evidence for the presence of Ca_V_1 or L-type channels in T cells (9). In excitable cells, Ca_V_1 channels require high voltage activation and have slow current decay kinetics. They are sensitive to 1,4-dihydropyridines (DHPs), drugs that include examples such as the L-type Ca^2+^ channel activator Bay K 8644 and the inhibitor nifedipine (8, 100).

Various pharmaceutical studies have assessed the relationship between Ca_V_1 channels and T cell Ca^2+^ signaling (15, 101, 102). The DHP antagonist nifedipine was shown to inhibit the proliferation of human T cells or peripheral blood monocuclear cells, or block the increase in intracellular Ca^2+^ following stimulation with mitogens (103–105). Kotturi et al. demonstrated that treatment of Jurkat T cells and human peripheral blood T cells with the DHP agonist Bay K 8644 led to an increase in intracellular Ca^2+^ levels and induced ERK 1/2 phosphorylation, while treatment with the DHP antagonist nifedipine blocked Ca^2+^ influx, ERK 1/2 phosphorylation, NFAT activation, IL-2 production, and T cell proliferation (15). It is interesting to note that DHPs can also have an effect on the function of K^+^ channels at micromolar concentrations; thus, conclusions drawn from these pharmaceutical studies (8, 15, 101, 102) regarding the contribution of Ca_V_1 channels to T cell function have undergone critique (106, 107). However, inhibitory effects were observed when DHP antagonists were used at concentrations well below those known to influence K^+^ channels (15, 108) as well as with calciseptine, a more specific blocker against Ca_V_1 channels that was also shown to hinder T cell Ca^2+^ influx (109, 110).

Many studies have established that Ca_V_1 channels are found in T cells (9). The first Ca_V_1 channel identified in T cells was Ca_V_1.4 (15–17), which is encoded by the *CACNA1F* gene initially cloned from the human retina (111) where Ca_V_1.4 facilitates Ca^2+^ entry into the photoreceptors and plays a role in tonic neurotransmitter release (112). Kotturi et al. demonstrated that Ca_V_1.4 mRNA and protein are found in Jurkat T cells and human peripheral blood T cells (15, 16). It was shown through sequence analysis that Ca_V_1.4 is expressed in human T cells as Ca_V_1.4a and Ca_V_1.4b, two novel alternative splice variants that are distinct from retina Ca_V_1.4 (16). Exons 31, 32, 33, 34, and 37 are missing in splice variant Ca_V_1.4a, leading to deletions of motif IV transmembrane segments S3, S4, S5, and half of S6. Consequences of these deletions include the removal of the voltage sensor domain and part of the DHP binding site and EF-hand Ca^2+^ binding motif. The absence of the voltage sensor may affect the channel’s voltage-gated activation, and partial deletion of the DHP binding site may diminish the sensitivity of T cell-specific Ca_V_1.4 channels, hence the need for large doses of DHP antagonists in order to fully impede Ca^2+^ influx through Ca_V_ channels in T cells (105). The splicing led to a frameshift that turned the C-terminus into a sequence that had 40% similarity to the Ca_V_1.1 channel found in skeletal muscle (16). In contrast, exons 32 and 36 are not found in splice variant Ca_V_1.4b, which results in a deletion of the extracellular loop between S3 and S4 as well as a partial deletion of transmembrane segment S6 in motif IV. In addition, the presence of an early stop codon in Ca_V_1.4b results in a prematurely truncated channel. The voltage sensing motif is still present, although it has been suggested that loss of the extracellular loop may influence the voltage sensing function of this channel (16). The S4 voltage sensor domain moves in response to membrane depolarization, and this splicing event may leave the domain in a conformation that prevents S4 movement (113, 114). These changes may provide a reason for the insensitivity of T cell Ca_V_1 channels to be activated by cell depolarization and alternatively, gating of this channel may be mediated via other mechanisms including TCR signaling or depletion of ER stores. Interestingly, Jha et al. showed that Ca_V_1.4 was localized to lipid rafts in the murine T cell plasma membrane. It was determined that Ca_V_1.4 was associated with T cell signaling complex components (115). These results suggest that the activity of Ca_V_1 channels could be directed in T cells by downstream TCR signaling events.

The role of Ca_V_ channels in T cell function has been examined in various *in vivo* studies. Mice with targeted deletions in the regulatory β subunits that regulate Ca_V_ channel assembly, plasma membrane targeting, and activation have been characterized (99, 116). Naïve CD4^+^ T cells express β3 and β4 family members, and their expression levels are increased in activated T cells. Following TCR cross-linking, CD4^+^ T cells from β3 or β4-deficient mice exhibit disrupted Ca^2+^ influx, cytokine secretion, and NFAT nuclear translocation (116). β4-deficient T cells have lower expression of Cav1.1, indicating a potential role for Ca_V_1 in lymphocyte function (116). CD8^+^ T cell populations in a β3-deficient mouse model have also been evaluated (115). Lower numbers of CD8^+^ T cells have been observed in **β*3^-/-^* mice, likely attributed to increased spontaneous apoptosis provoked by greater Fas expression. During activation, these CD8^+^ T cells show decreased Ca^2+^ entry, NFAT nuclear translocation, and proliferation. It was established that β3 associates with Ca_V_1.4 and various TCR signaling proteins, implying its role in TCR-gated Ca^2+^ signaling (115). When the AHNAK1 protein, a scaffold protein needed for surface expression of Ca_V_1.1, was disrupted, T cells demonstrated a reduction in NFAT activation and Ca^2+^ influx that translated to impaired effector function (117, 118). Studies have also started to approach the topic of differential Ca^2+^ signaling in T cell subsets (119, 120), providing evidence that Ca_V_1.2 and Ca_V_1.3 are expressed in Th2 but not Th1 differentiated effector T cells. When Ca_V_1.2 and/or Ca_V_1.3 expression was knocked down in Th2 cells with antisense oligodeoxynucleotides, this led to hindered Ca^2+^ influx following TCR stimulation and cytokine secretion, as well as impaired ability to induce asthma in an adoptive transfer model (119). In order to increase our understanding of differences in Ca^2+^ responses, further analysis is required with respect to differential expression of Ca_V_1 channel subtypes and of their splice variants.

A Ca_V_1.4-deficient mouse model (121) was used by Omilusik et al. to clearly demonstrate a T cell-intrinsic role for Ca_V_1 channels in the maintenance, survival, and activation of naïve CD4^+^ and CD8^+^ T cells *in vivo*. It was demonstrated that Ca_V_1.4 is required for TCR-induced regulation of free Ca^2+^ in the cytosol and downstream TCR signaling, affecting induction of the Ras/ERK and NFAT pathways, IL-7 receptor expression, and IL-7 response. Ca_V_1.4 deficiency resulted in defective immune responses when exposed to the model bacteria *Listeria monocytogenes* (17). It seems as though Ca_V_1.4 may operate to create intracellular Ca^2+^ stores in the ER. Low-level TCR signaling through interactions with self-antigens (i.e., self-peptides/self-MHC molecules) may result in Ca_V_1.4-mediated Ca^2+^ influx from outside the cell, allowing the filling of intracellular stores and the initiation of a pro-survival program. This recent data supports the concept that in the absence of Ca_V_1.4, there is a reduction in the influx of extracellular Ca^2+^ coupled to self/MHC-TCR interaction, resulting in low cytoplasmic Ca^2+^ levels and depleted Ca^2+^ ER stores (17). In the proposed model, following TCR stimulation of Ca_V_1.4-deficient T cells, there is an impaired Ca^2+^ release from the ER due to reduced levels of stored Ca^2+^, diminished subsequent SOCE, and decreased Ca^2+^ influx through CRAC channels resulting in dampened Ca^2+^-dependent signaling. The lack of Ca_V_1.4 results in an inability of naïve T cells to thrive and maintains a state of immunological activation and exhaustion (17), which may provide an explanation for many of the observations regarding the role Ca_V_ channels play in T cells. In general, knock-outs of Ca_V_1 channel components in T cells have more severe phenotypes compared to those of other Ca^2+^ channel families in T cells. This clearly supports the concept that Ca_V_1 channels are important in the regulation of T cell biology.

**Figure 1:**
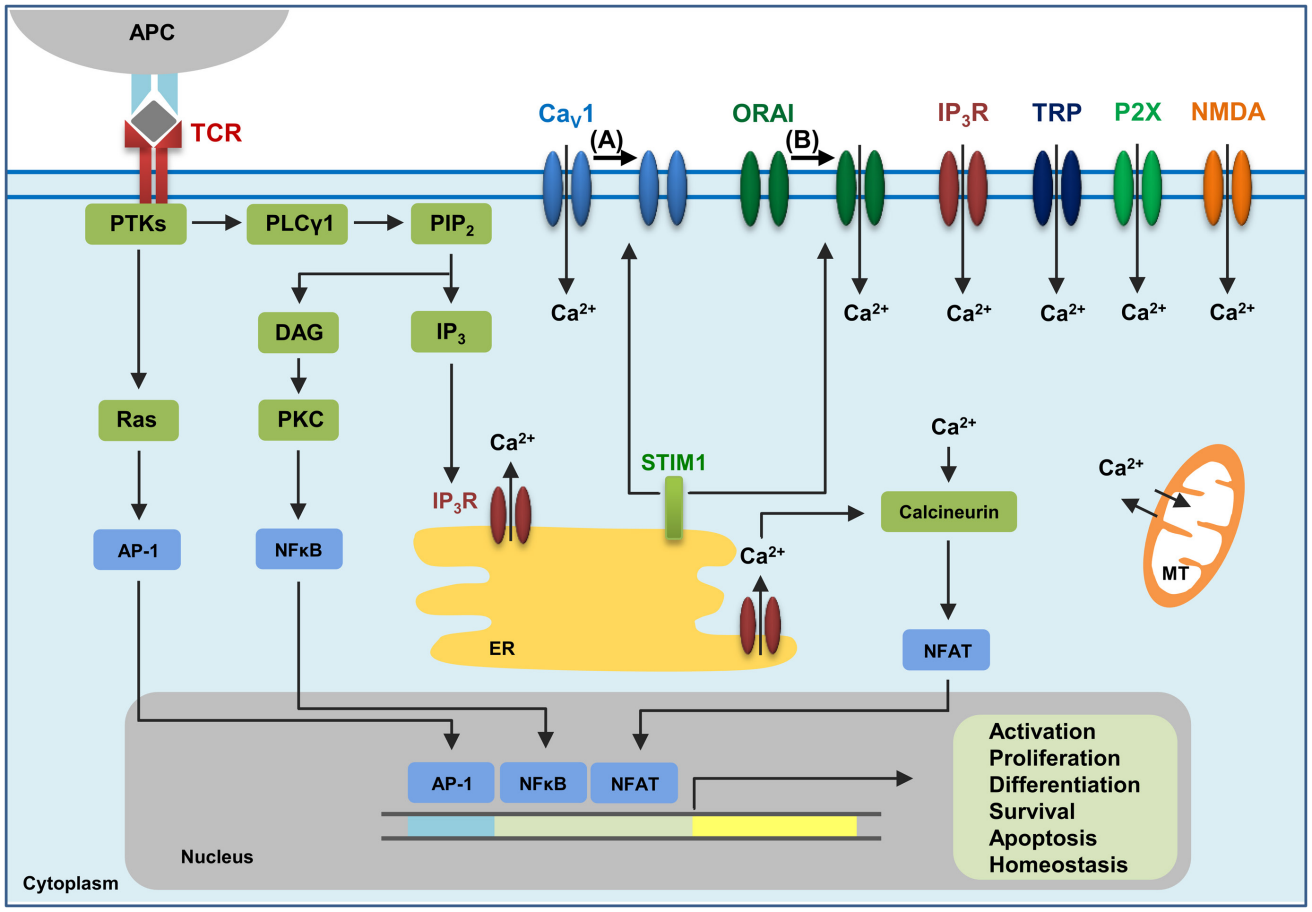
**The calcium channels in T cells**. T cell receptor (TCR) engagement by a peptide-MHC on an antigen presenting cell (APC) induces protein tyrosine kinases (PTKs) to activate phospholipase Cγ1 (PLCγ1), which cleaves phosphatidylinositol 4,5-bisphosphate (PIP_2_) from plasma membrane phospholipids to generate diacylglycerol (DAG) and inositol-1,4,5-trisphosphate (IP_3_). Elevated levels of IP_3_ in the cytosol lead to the release of Ca^2+^ from IP_3_Rs located in the endoplasmic reticulum (ER). Ca^2+^ depletion from the ER induces Ca^2+^ influx from the extracellular space through the plasma membrane channel, ORAI1. Several additional channels also operate during TCR-mediated Ca^2+^ signaling. These include plasma membrane IP_3_R activated by the ligand IP_3_, transient receptor potential (TRP) channels that can be operated by DAG and SOCE, adenosine triphosphate (ATP)-responsive purinergic P2 (P2X) receptors, glutamate-mediated *N*-methyl-d-aspartate activated (NMDA) receptors, and voltage-dependent Ca^2+^ channels (Ca_V_) that may be regulated through TCR signaling events. The mitochondria (MT) also control cytoplasmic Ca^2+^ levels. Increase in intracellular Ca^2+^ results in activation of calmodulin–calcineurin pathway that induces NFAT nuclear translocation and transcription of target genes to direct T cell homeostasis, activation, proliferation, differentiation, apoptosis and survival. Within this complex network of Ca^2+^ signaling, a model of the reciprocal regulation of Ca_V_1 and ORAI1 in T cells has been proposed. (A) Low-level TCR signaling through interactions with self-antigens (i.e., self-peptides/self-MHC molecules) may result in Ca_V_1 (particularly Ca_V_1.4) activation and Ca^2+^ influx from outside the cell. This allows for filling of intracellular Ca2+ stores and initiation of a signaling cascade to activate a pro-survival program within the naïve T cell. STIM1 is not activated in this scenario and, consequently, ORAI1 remains closed. (B) Strong TCR signaling through engagement by a foreign peptide-MHC induces the downstream signaling events that result in ER Ca^2+^ store depletion and STIM1 accumulation in puncta in regions of the ER near the plasma membrane allowing interactions with Ca^2+^ channels. ORAI1 enhances STIM1 recruitment to the vicinity of Ca_V_1 channels. Here, STIM1 can activate ORAI1 while inhibiting Ca_V_1.

There is increasing evidence in support of relationships between different types of Ca^2+^ channels, and one such relationship is the one between Ca_V_1.2 and ORAI1 (122, 123). Following Ca^2+^ store depletion in the ER, STIM1 oligomers form at ER-plasma membrane junctions, thereby allowing the STIM1 CRAC-activating domain (CAD) to interact with the C-terminus of ORAI1 and Ca_V_1.2 channels. STIM1 activates ORAI1 channels, which then open resulting in sustained Ca^2+^ entry from the extracellular space. On the other hand, STIM1 blocks Ca^2+^ influx through Ca_V_1.2 and promotes its internalization (122, 123). It may be possible that strong TCR signaling via engagement by a foreign peptide-MHC may lead to this activation of ORAI1 and inhibition of Ca_V_1 channels (Figure 1). In contrast, weaker TCR signaling through engagement with self-antigens might not elicit STIM1 to localize to the plasma membrane, hence activating Ca_V_ and blocking ORAI1. Further investigation regarding the interplay between different Ca^2+^ channel families will greatly advance the field of Ca^2+^ channel research.

In conclusion, while the specific functions of the various Ca^2+^ channels discussed in this review have yet to be fully explored, these channels clearly play important roles in T cell biology and may serve as useful targets for therapeutic drugs. Many drugs already exist for modifying Ca_V_1 channels, for example, isradipine and propofol. Moving forward, drugs that target specific Ca^2+^ channel splice variants found in lymphocytes may act as superior immunomodulatory agents compared to what are currently available. Pertinent applications would likely include treatment of autoimmune diseases, reduction of transplant rejection risk, and treatment of a wide range of other conditions requiring modulation of the immune system.

## Conflict of Interest Statement

The authors declare that the research was conducted in the absence of any commercial or financial relationships that could be construed as a potential conflict of interest.
